# Supplementary material for: Synergy, Additivity and Antagonism between Esculetin and Six Commonly Used Chemotherapeutics in Various Malignant Melanoma Cell Lines—An Isobolographic Analysis
Source: Molecules. 2023 May 5;28(9):3889. doi: 10.3390/molecules28093889 (PMC10180450; doi:10.3390/molecules28093889)
Supplement: Supplementary file 1 [file molecules-28-03889-s001.zip › molecules-2345472-supplementary.pdf]

Supplementary Materials

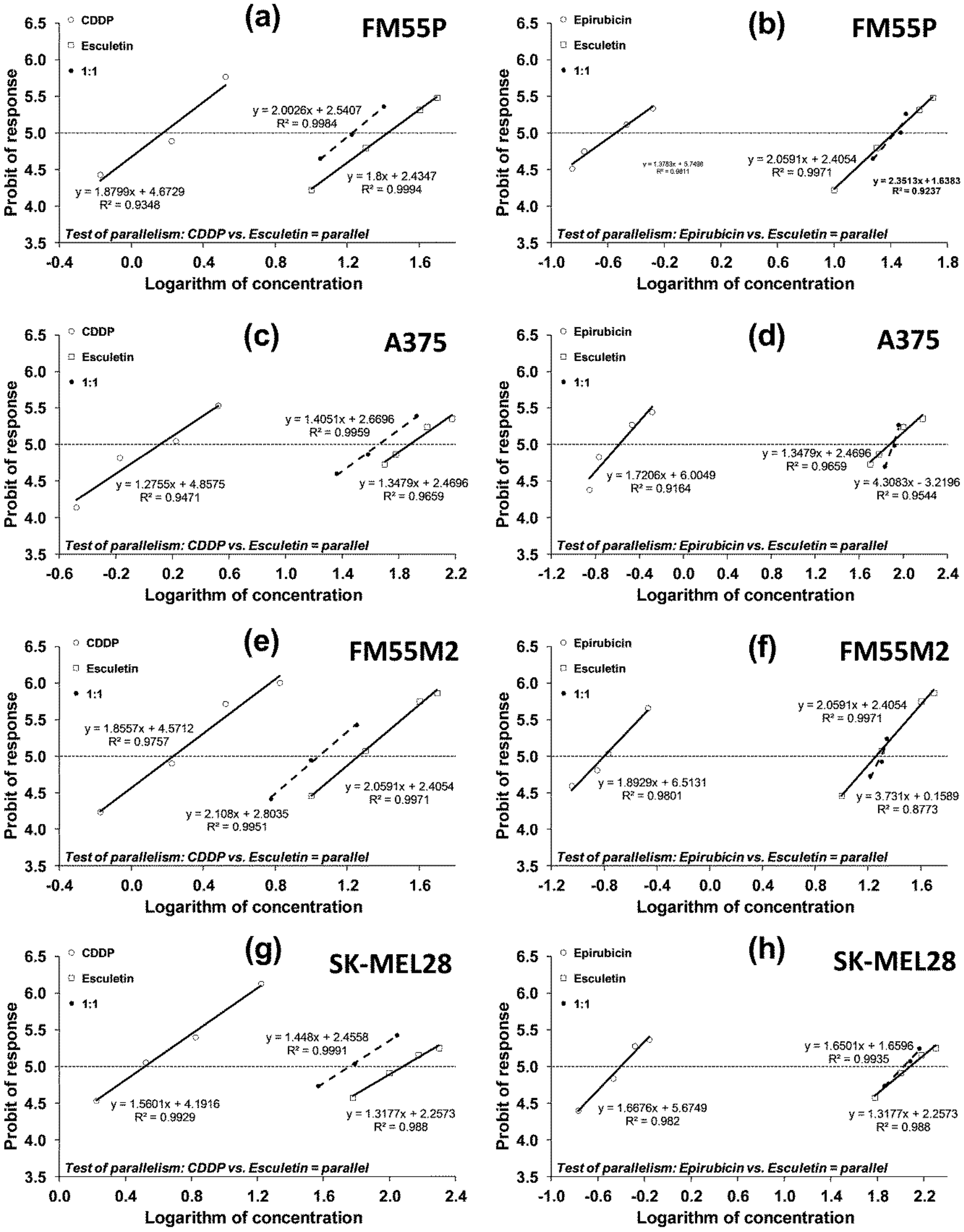

Figure S1. Concentration–effect lines for esculetin and cisplatin (a,c,e,g), esculetin and epirubicin (b,d,f,h) administered alone and in combination in the fixed-ratio of 1:1, illustrating the anti-proliferative effects of the drugs in the malignant melanoma cell lines: FM55P (a,b), A375 (c,d) FM55M2 (e,f) and SK-MEL28 (g,h) measured in vitro by the MTT assay.

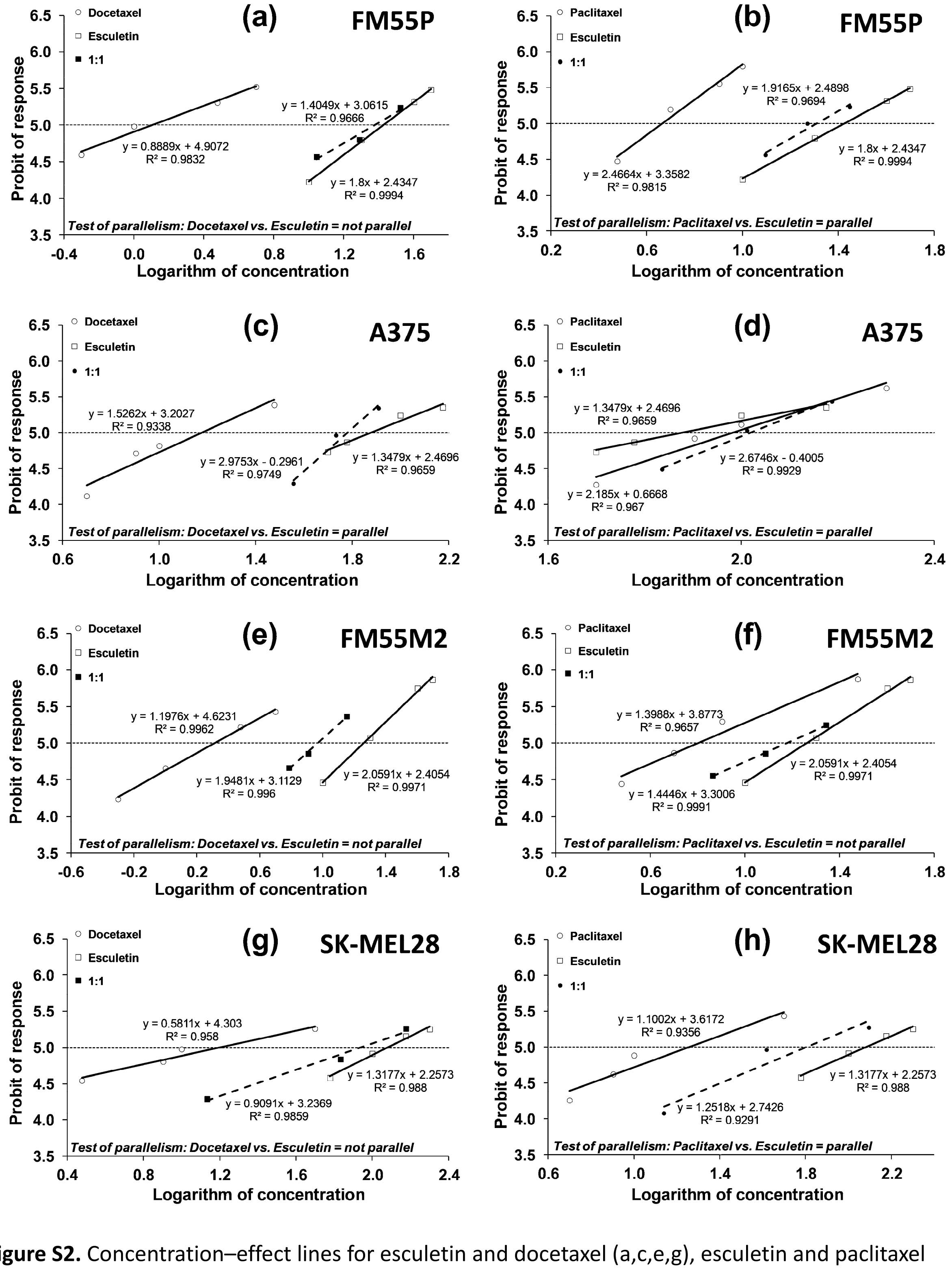

Figure S2. Concentration–effect lines for esculetin and docetaxel (a,c,e,g), esculetin and paclitaxel (b,d,f,h) administered alone and in combination in the fixed-ratio of 1:1, illustrating the anti-proliferative effects of the drugs in the malignant melanoma cell lines: FM55P (a,b), A375 (c,d) FM55M2 (e,f) and SK-MEL28 (g,h) measured in vitro by the MTT assay.

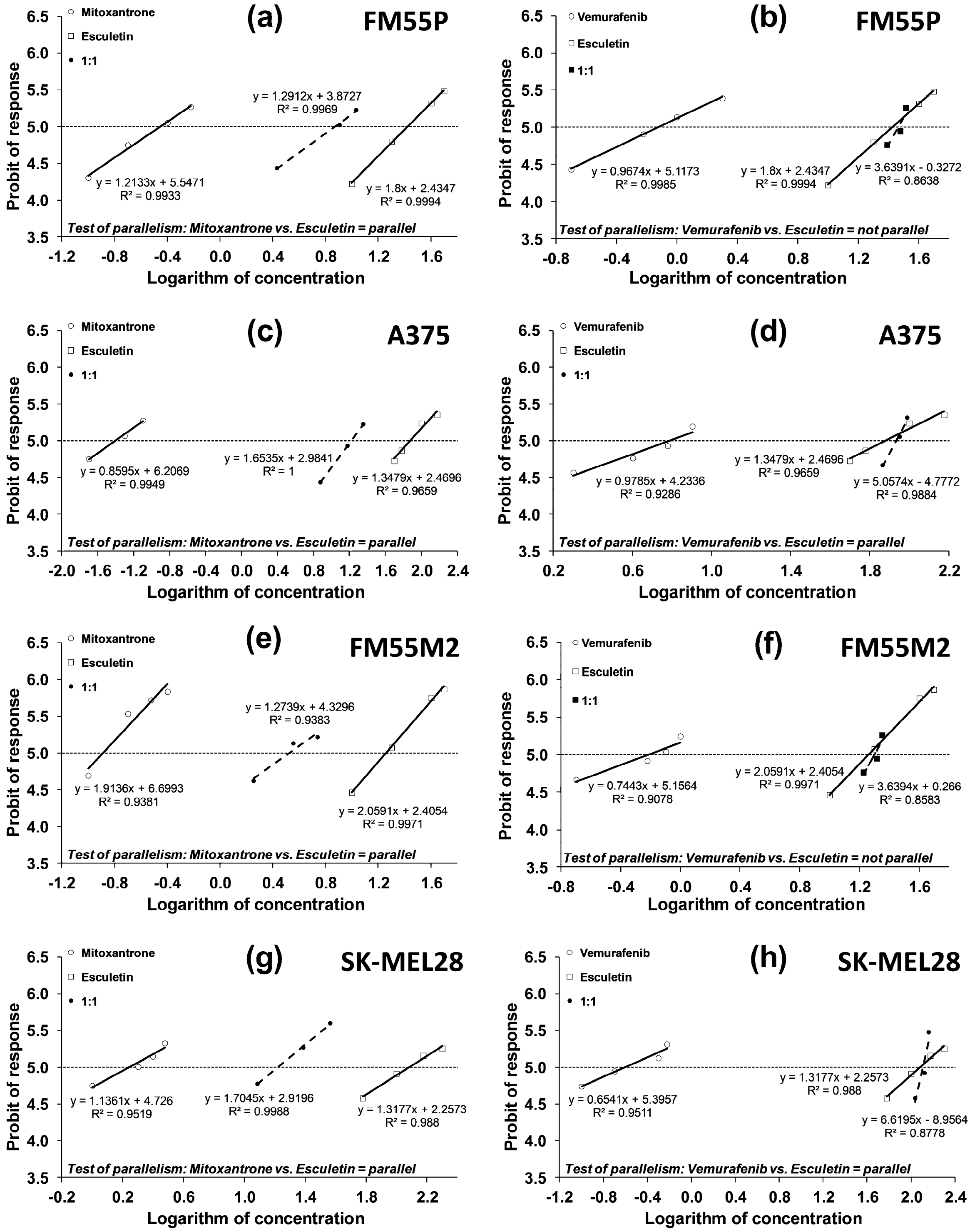

Figure S3. Concentration–effect lines for esculetin and mitoxantrone (a,c,e,g), esculetin and vemurafenib (b,d,f,h) administered alone and in combination in the fixed-ratio of 1:1, illustrating the anti-proliferative effects of the drugs in the malignant melanoma cell lines: FM55P (a,b), A375 (c,d) FM55M2 (e,f) and SK-MEL28 (g,h) measured in vitro by the MTT assay.
